# Physicochemical, Microstructural, and Rheological Characterization of Tigernut* (Cyperus esculentus)* Starch

**DOI:** 10.1155/2019/3830651

**Published:** 2019-06-02

**Authors:** P. T. Akonor, C. Tortoe, C. Oduro-Yeboah, E. A. Saka, J. Ewool

**Affiliations:** ^1^Food Technology Research Division, Council for Scientific and Industrial Research-Food Research Institute, P.O. Box M20, Accra, Ghana; ^2^Department of Animal Biology and Conservation Sciences, University of Ghana, Legon, Ghana

## Abstract

The aim of this study was to characterize the physicochemical properties of starch isolated from two varieties of tigernuts. The results showed wide variations between the two types of tigernuts. Mean granule sizes were 11.1 and 6.1 *μ*m, respectively, for starch from the yellow and black while amylose content ranged from 19 to 21%. Starch gels from the yellow variety were more stable to freeze-thaw and recorded 37.1% syneresis, compared to 56.5% after the first storage cycle. Pasting properties were significantly different (*p *< 0.05) among starch from the two tigernut varieties, with black recording higher peak viscosity, lower breakdown, and higher setback viscosity. Gels made from the yellow variety were clearer, softer, more adhesive, and more cohesive. Both gels showed a pseudoplastic flow behavior without thixotropy.

## 1. Introduction

*Cyperus esculentus* is an edible grass plant which produces nut-like tubers known as tigernut. The nuts, which are about 30 mm long (Parker et al., 2000), are characterized by a sweet and somewhat milky taste. It is cultivated and consumed in many tropical and subtropical countries. Two major varieties (black and yellow) have been identified in many growing areas.* C. esculentus* has significant levels of major storage nutrients as well as micronutrients. The tuber mainly contains carbohydrates (more than 60%), which to a large extent consists of starch and dietary fiber. Lipids are the second most dominant constituent (25%), while protein and ash make up 5% and 1.7% of the nuts, respectively [1]. Even though it largely remains underutilized, its application in certain food products has been documented. For instance, its use in the production of bread, beverages, and burgers has, respectively, been reported by Aguilar, Albanell, Minarro, Guamis, and Capellas [2], Kizzie-hayford, Jaros, Schneider, and Rohm [3], and Sánchez-Zapata et al. [4]. Additionally, its application in the pharmaceutical industry has also been explored [5].

Tigernut is a very useful source of starch. Starch is a key component of most tuber and cereal crops, and it essentially consists of two glucose polymers: amylose and amylopectin. These polymers are contained in varying amounts, depending on the source of starch. Native starch is generally known to be composed of up to 25% amylose and 75% amylopectin. Whereas amylose is generally linear with glucose units linked by *α*-1,4 glycosidic bonds, amylopectin is highly branched and therefore is much bulkier. These two components ultimately account for the behavior of starches in food systems.

As described by Manek, Builders, Kolling, Emeje, and Kunle [6], tigernut starch is odourless and has a white-off white appearance. Its flow properties are comparable to maize and potato starch [7]. This suggests that tigernut starch possesses good physical qualities and therefore has a great potential for use in a wide variety of food and nonfood applications. However, the properties of starch from the major varieties (black and yellow) of the crop have not been widely studied and characterized. This restricts the utilization of tigernut starch in the manufacture of food and other industrial products. In this study, starch isolated from the two major cultivars of tigernuts was characterized in order to understand its behavior and functionality in food systems.

## 2. Materials and Methods

### 2.1. Tigernuts

The two major cultivars of tigernut (black and yellow) were used in the study. The tigernuts were fully matured and freshly harvested. They were cleaned of adhering earth material, washed in clean potable water, and transported to the laboratory for starch extraction and analysis.

### 2.2. Starch Isolation

Tigernut starch was isolated following the method of [8], with slight modifications. Tigernut tubers were twice washed in potable water before soaking in water overnight. The soak water was drained and the tubers were milled into a slurry in a laboratory blender (Waring E8420, Torrington, USA) using water to aid the process. The slurry was strained through a cheese cloth and the filtrate was left to stand for 4 h. Thereafter the supernatant was discarded and the starch layer was resuspended in distilled water and filtered through a 150 *μ* mesh. The starch was allowed to settle after standing for 2 h and the supernatant was discarded. The procedure of resuspending, filtration (100 *μ* mesh) settling was repeated once again before the isolated starch was dried in an air oven at 40°C for 18 h. The dried starch was milled into fine powder and sealed air-tight in HDPE bags for analyses.

### 2.3. Optical Properties

#### 2.3.1. Color

Objective color of starches from the 2 major varieties of tigernut was measured using a Minolta-CR410 Chromameter (Minolta, Japan). The instrument was calibrated against a standard white tile (L_0_ = 97.63, a_0_ = 0.31, and b_0_ = 4.63) before use. Starch samples were contained in a transparent pyrex petri dish and covered with same. Starch color was described using in L*∗* C*∗* h notation.

#### 2.3.2. Paste Clarity

Starch paste clarity was determined according to Craig, Maningat, Seib, and Hoseney [9]. Five milliliters of 1% starch suspension in 15 mL screw-capped centrifuge tubes was incubated in a boiling water bath for 30 min, with continual shaking. The starch solution was cooled to room temperature and its transmittance (%) measured against a water blank at 650 nm on a UV-VIS spectrophotometer (Cecil Instruments, England).

### 2.4. Starch Content

#### 2.4.1. Amylose and Amylopectin Determination

Amylose content of tigernut starches was determined following the method described by Zhu et al. [8]. One hundred milligram of starch was weighed into a 100 mL standard bottle and washed with 1 mL of 95% ethanol, followed by 9 mL of 1 N NaOH. The suspension was heated in a boiling water bath for 15 min, with continual shaking. Thereafter the solution was allowed to cool to room temperature and made up to the mark with distilled water. An aliquot (5 mL) of this solution was pipetted into a separate 100 mL standard bottle; 1 mL of 1 M acetic acid was added followed by 2 mL of iodine solution (0.2 g I + 2 g KI in 100 mL solution), before making up to the mark with distilled water. The resulting solution was mixed thoroughly and allowed to stand for 20 min for color development. The absorbance of the solution was measured at 620 nm using a UV-VIS spectrophotometer (Cecil Instruments, England).

### 2.5. Swelling and Solubility Indices

An amount (150 mg) of starch was weighed into a centrifuge tube and 10 mL of distilled water added [10]. The suspension was vortexed and incubated at 85°C for 30 min in a water bath equipped with a shaker (Grant OLS 200, England). The samples were cooled to room temperature and centrifuged at 2000 x g for 35 min and the supernatant was dried to constant weight in an air oven at 105°C, while the sediment was weighed directly. The swelling power and water solubility index were determined using the relations:

(1) SP=wt  of  precipitated  paste  Wpwt  of  sample  (Wo)−wt  of  residue  in  supernatant  WrSI=wt  of  residue  in  supernatant  Wrwt  of  sample  Wo×100

### 2.6. Syneresis

The procedure described by Simi and Abraham [11] was used to determine the freeze-thaw stability. Briefly, a 6% starch suspension was held at 95°C for 15 min in a water bath (Grant OLS 200, England), cooled to 50°C, and kept at this temperature for 15 min. Aliquots of 50 mL were sampled into centrifuge tubes and kept at 4°C and -18°C for 18 h. Thereafter, samples were centrifuged at 604 x g for 10 min and the amount of water separated from the starch gel was measured. Syneresis (%) was computed using the following formula:

(2) $$\begin{array}{c} Syneresis=\frac{\text{wt of supernatant}}{\text{wt of sample}}\times 100 \end{array}$$

### 2.7. Flow Behavior of Starch Gels

Starch flow behavior was measured with a DV2T Viscometer (Brookfield Engineering Inc.). The viscosity of starch gels (5% starch heated at 95°C for 30 min with continual shaking and cooled to room temperature) was determined at different spindle speeds (5-25 rpm) using an RV spindle 2, without the guard leg. Using an end condition of 10 points, viscosity data was recorded at 2 min intervals. Measurements were taken in a 250 mL beaker, 2 min after spindle was immersed in the starch gel. This was done in order to allow thermal equilibrium between test sample and spindle and to eliminate the effect of immediate time dependence, as directed by the manufacturer.

### 2.8. Pasting Properties

Starch pasting characteristics were quantitatively determined on a 10% slurry, using the Brabender Viscoamylograph (Brabender Instruments Inc., Duisburg, Germany). Viscosity profile indices recorded were peak viscosity, pasting temperature, hot paste viscosity, cool paste viscosity, breakdown, and setback viscosity.

### 2.9. Gel Texture Analysis

Texture Profile Analysis (TPA) was performed on starch gels from the pasting analysis using a Texture Analyzer (TA.XT*plus*, Stable Microsystems, Surrey, UK) with a compression cylindrical probe. Gels from the pasting experiments were kept at 4°C overnight before the TPA. A double bite compression cycle with the probe set to compress starch gels to about 75% of its height at a test speed of 1 mm/s during each cycle was used. Hardness of the gels was determined as the peak force required to compress it through 75% of its height. Adhesiveness, springiness, and cohesiveness of starch gels were also derived from the Exponent software.

### 2.10. SEM Imaging

Granular morphology was characterized by SEM (JEOL JSM-6390, Tokyo, Japan). Starch samples were first coated with platinum before imaging at a voltage of 3.0 kV and X1200 magnification.

### 2.11. Statistical Analysis

All the analyses were done in triplicate. T-test was used to compare the means obtained from the two cultivars (Minitab 17.0.1).

## 3. Results and Discussions

### 3.1. Microstructure of Tigernut Starch

**Figure 1:**
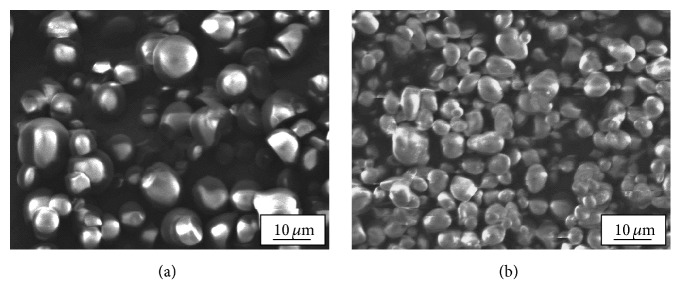
SEM images of starch granules isolated from yellow (a) and black (b) tigernuts.

Micrographs of starch granules show that the yellow cultivar had fairly equal amounts of loosely packed small and large granules, whereas starch from the black cultivar was dominated by small granules which were rather clustered and densely packed (Figure 1). Generally, the starch granules were within the size range of small and medium [12]. Granule diameters ranging between 5.5 to 16.6 *μ*m (mean 11.1 *μ*m) and 3.3 to 12 *μ*m (mean 6.1 *μ*m) were, respectively, recorded for starch from yellow and black cultivars. Clearly, both starches largely consisted of spherical granules with smooth surfaces but black contained a fairly large number of smaller granules. Some oval shape types measuring nearly 10 *μ*m on its major axis (black) and damaged granules and a few with faceted sides (yellow) were seen in the tigernut starches. Even though their granules were relatively smaller, the shapes and surface features of these tigernut starches closely resembled starches from other tropical tubers such as sweetpotato [8].

### 3.2. Physicochemical Properties of Tigernut Starches

The apparent amylose content of the tigernut starches ranged between 19 and 21% (Table 1). Although this margin was slim, T-test showed a significant difference (*p* < 0.01) between amylose content of the two starches. The results suggest that products made from these starches may vary in some key attributes. This is because relative proportion of amylose and amylopectin is largely responsible for the functional behavior and nutritional properties of starches and starch-based food products. For instance, high amount of amylose enhances film-forming ability, slows digestibility, and results in higher expansion ratios in extruded products [13] and compact and less open-structured bread crumb [14].

This range observed in the present study was consistent with the amylose content of native starches (15-30%) noted by Bertoft [15], 17.9 – 23.6% reported for cassava by Defloor, Dehing, and Delcour [16], and 10.1 – 20.2% for sweetpotatoes [17]. That notwithstanding, the amylose was lower than starches from some tropical root crops such as cocoyam and yam (26.7%) [18].

The ability of starch to swell and eventually produce a paste when heated in water is one of its essential features. Granule swelling is generally attributed to amylopectin, whereas amylose plays the role of a diluent and also restricts swelling [19]. Starch isolated from the yellow cultivar had the highest swelling power (SP), water solubility index (WSI), and water binding capacity (WBC). The SP is an indicator of how much water one gram of starch can absorb at a given temperature, while SP represents the percentage of leached AM and AP at this same temperature [20]. The trends observed in the results of SP and SI may be ascribed to differences in amylose content of the two starches. Significantly (*p* < 0.05) higher amounts of AM in the black cultivar resulted in lower SP compared to the yellow cultivar. Since AP is postulated to leach out at temperatures beyond 90°C [21] and 85°C was use in this study, it may be assumed that SI was largely due to leached AM, rather than AP. Although the black cultivar had a higher AM content, granule size and morphological features may account for its rather lower SI. The association of AM and AP plays a key role in WBC of starches, with loosely associated polymers being responsible for higher WBC. Also, differences in WBC could have resulted from variations in the degree of available water binding sites, which is controlled by ultrastructural and compositional differences in the two starches [22].

### 3.3. Optical Properties of Tigernut Starch

Optical properties of the tigernut starches were described by their color and paste clarity. Color plays a key role in raw material selection for food processing as well as meeting consumer expectation in finished products. The results showed that the two starches had an identical °Hue (*p* > 0.05) but starch of the yellow cultivar was obviously the whiter and brighter of the two (Table 2). Chroma values, which indicate the extent of color saturation, ranged between 95 and 102 for the two starches, whereas an average hue of 4.2° was recorded.

Starch paste clarity is an essential property of starch gels, especially for industrial uses, and varies considerably among different botanical sources. As hypothesized by Craig, Maningat, Seib, and Hoseney [9], swollen starch granules allow light to pass through them (instead of being reflected) because their ability to reflect light weakens as starch molecules dissociate. This phenomenon is responsible for clarity or opacity of starch gels. Consequently, the presence of granule remnants and the interaction between leached materials cause high opacity. Additionally, factors that restrict swelling and dispersibility, such as amylose content and its conformation, lipids, and crosslinking negatively affect the clarity of starch gels, whereas the presence of sucrose improves clarity. Generally, while starches from tuber crops (e.g., cassava and potato) form transparent gels, gels from cereals such as maize and wheat are opaque. Clarity of gels made from the two starches was significantly different (*p* < 0.05). Gel from the yellow variety recorded a higher transmittance and was therefore the most transparent of the two, probably as a result of its lower amylose content and larger granule size [23]. This makes it a better choice for use in food products such as soups and dessert powders, where clarity is required.

### 3.4. Freeze Thaw Stability of Tigernut Starch

**Figure 2:**
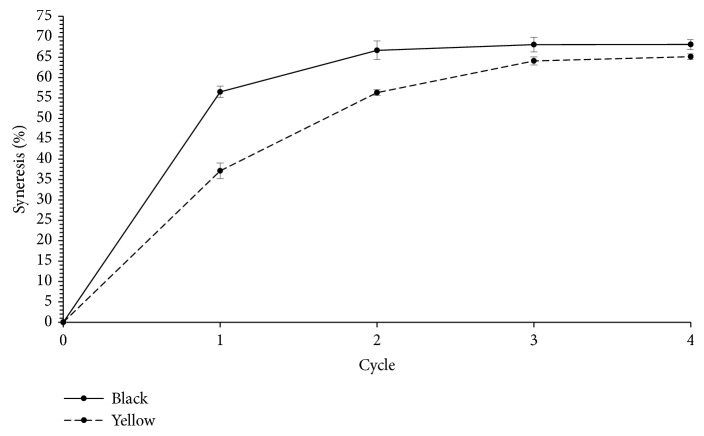
Freeze-thaw stability of starch gels from yellow and black tigernuts.

The freeze-thaw behavior of gels from the two varieties of tigernuts was typical of most native starches of root and tuber origin (Figure 2). This property commonly refers to the ability of starch to withstand unnecessary physical changes during freezing and thawing [24] and it particularly affects products that require low temperature storage. Yellow tigernut starch gel exhibited a better freeze-thaw stability (slower and lower phase separation) with a final exudate of 65% of the initial weight of the gel, compared to the black variety (68%). Generally, as established elsewhere [24], syneresis correlates with starch's propensity to retrograde. The differences in the gels' syneresis, in this study, may have resulted from varying amylose content among the two cultivars, with higher amylose content showing a stronger tendency to retrograde. The lower syneresis value of the yellow variety may indicate slower retrogradation of the gel because of strong interactions between dispersed amylose-amylopectin and water molecules [25]. Similarly, in other root crops such as potato, tapioca, and arrowroot, high amounts of water exuded from their starch gels during freeze-thaw cycles [26].

### 3.5. Rheological Properties of Tigernut Starch

The pasting pattern of tigernut starches was typical of many root and tuber starches, which are characterized by high peak and breakdown viscosities. Marked differences were observed in the pasting patterns of starches from the two cultivars (Table 3). For instance, the starch from yellow cultivar began to thicken at 67°C compared to swelling in the black cultivar, which occurred at 71°C. This observation suggests that granules of the yellow tigernut starch swell freely and would require lower energy and, by extension, less time to cook. The free swelling nature of the yellow cultivar was evident in its higher swelling capacity (Table 1). The pasting temperature in this study was lower than the range (75 - 79°C) earlier reported by Manek et al. [6]. Whereas wide variations were noticed in the other pasting properties, a narrow range (879 - 866 BU) of PV was observed among the two cultivars of tigernuts. The black cultivar, which contained smaller starch granules, had a higher peak viscosity. Considering granule size, the results observed were in contrast with Noda et al. [27], where authors associated smaller granule size with lower peak viscosity and breakdown in potato starch. Eliasson and Karlsson [28], however, suggest that particle size distribution may also affect gelatinization properties of starch. The peak viscosities were comparable to cassava starch (635-920 BU) as reported by Asaoka, Blanshard, and Rickard, [29]. The black tigernut cultivar had a higher holding strength (lower BD viscosity) and therefore proved to be the most stable of the two pastes. This paste also had the highest SB viscosity. Setback occurs during the cooling cycle and is characterized by an increase in viscosity as a result of the reassociation of amylose molecules through H-bonding [30]. This explains why black cultivar, which had a higher amylose content, subsequently had higher CPV and SB. High SB correlates with high syneresis and indicates a higher propensity of starch retrogradation.

**Figure 3:**
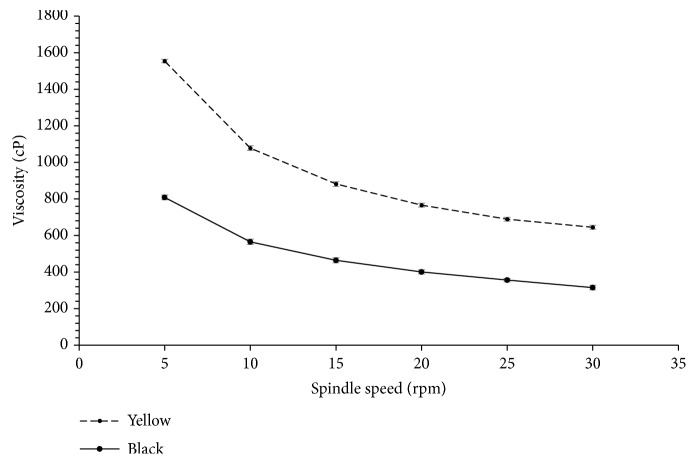
Viscosity profile of gels from yellow and black tigernut starch.

The texture of the two gels varied widely. Hardness, adhesiveness, and cohesiveness of the gels made from the two starches were significantly different (*p* < 0.05) as shown in Table 3. The starch gel made from yellow cultivar was softer, more adhesive, and more cohesive compared to starch gel made from the black cultivar. These differences may be explained by differences observed between amylose content and granule sizes of the starch. As noted by Mua and Jackson [31], starches with higher amylose content and longer amylopectin chains may produce harder starch gels. Furthermore, gel firmness may occur as a result of retrogradation, and this is associated with syneresis of water and crystallization of amylopectin [32]. This is confirmed by the association between hardness and the extent of syneresis in starch gels observed in this study. Simi and Abraham [11], working on rice starch gels, also noticed a relationship between texture properties and starch granule size. In their study, gels made from rice with bigger starch granules were softer, more adhesive, and more cohesive compared to those made from rice with smaller granule sizes.  Figure 3 presents the flow behavior of gels made from tigernut starches. The 5% starch gel showed a typical non-Newtonian behavior, which is characterized by changes in apparent viscosity due to changes in shear stress or shear rate [33]. A reduction in apparent viscosity with increasing shear rate was observed, and this indicates the pseudoplastic nature of the starch gels. Starch gels made from yellow tigernut were more viscous than those made from black tigernut. For instance, at the lowest shear rate (spindle speed), a viscosity of 808 cP and 1554 cP was recorded correspondingly to starch gels from yellow and black tigernut cultivars, respectively. Similar pseudoplastic flow behavior has been reported for sago starch gels [34].

## 4. Conclusion

The results showed wide variations in some physicochemical properties of starch isolated from the two tigernuts. Although both mainly consisted of spherical granules, the starch of the yellow cultivar had fairly larger granules compared to the starch of the black cultivar. Amylose content ranged between 19 and 21% for the two. Swelling and water solubility properties were significantly higher (*p* < 0.05) in starch isolated from the yellow cultivar. A narrow range (866-879 BU) of peak viscosities was observed in starch from the two cultivars. Retrogradation tendency was higher in black tigernut starch gel that made it harder and less cohesive among the two cultivars. Findings of the study suggest that starches from these two varieties of tigernut may be suited for different culinary or industrial purposes.

## Figures and Tables

**Table 1:** Physicochemical properties of starches from yellow and black tigernut cultivars.

Properties	Yellow	Black
Mean granular size (*μ*m)	11.1±1.0 ^b^	6.1±0.7^a^
Amylose (g/g)	19.1±0.1^a^	21.9±0.1 ^b^
Swelling power (g/g)	11.3±0.1^b^	9.9±0.1 ^a^
Water solubility index (%)	13.5±0.2^b^	11.6±0.3^a^
Water binding capacity (g/g)	186.9±1.1^b^	175.4±1.5^a^

Superscripts within the same column imply significant differences at *p* ≤ 0.05

**Table 2:** Optical properties of starch from two cultivars of tigernut.

Properties	Yellow	Black
Color		
L*∗*	92.5±0.3^b^	89.3±0.2^a^
C*∗*	95.1±0.1^a^	102.0±0.2^b^
H (°)	4.2±0.1^a^	4.3±0.1^a^
Paste clarity (%)	30.8±1.6^b^	14.7±1.2^a^

Superscripts within the same column imply significant differences at *p* ≤ 0.05

**Table 3:** Tigernut starch pasting and gel texture properties.

Properties	Yellow	Black
Pasting Temperature (°C)	66.5±0.5^a^	71.4±0.5^b^
Peak Viscosity (BU)	866.4±2.1^a^	879.3±0.9^b^
Hot Paste Viscosity (BU)	310.1±0.6^a^	421.3±1.2^b^
Cold Paste Viscosity (BU)	515.1±0.5^a^	783.1±1.4^b^
Breakdown (BU)	556.3±1.1^b^	458.2±2.0^a^
Setback (BU)	205.1±1.0^a^	363.2±3.5^b^
Hardness (g.force)	916.02±10.98^a^	2357.69±8.23^b^
Adhesiveness (g.s)	49.69±4.72^b^	6.83±0.34^a^
Cohesiveness	0.48±0.10^b^	0.40±0.14^a^

Superscripts within the same column imply significant differences at *p* ≤ 0.05

## Data Availability

The data used to support the findings of this study are included within the article.

## References

[1] Rubert J., Sebastià N., Soriano J. M., Soler C., Mañes J. (2011). One-year monitoring of aflatoxins and ochratoxin A in tiger-nuts and their beverages. *Food Chemistry*.

[2] Aguilar N., Albanell E., Minarro B., Guamis B., Capellas M. (2014). Effect of tiger nut-derived products in gluten-free batter and bread. *Food Science and Technology International*.

[3] Kizzie-hayford N., Jaros D., Schneider Y., Rohm H. (2014). Characteristics of tiger nut milk: effects of milling. *International Journal of Food Science and Technology*.

[4] Sánchez-Zapata E., Muñoz C. M., Fuentes E. (2010). Effect of tiger nut fibre on quality characteristics of pork burger. *Meat Science*.

[5] Builders P. F., Mbah C. C., Adama K. K., Audu M. M. (2014). Effect of pH on the physicochemical and binder properties of tigernut starch. *Starch/Stärke*.

[6] Manek R. V., Builders P. F., Kolling W. M., Emeje M., Kunle O. O. (2012). Physicochemical and binder properties of starch obtained from Cyperus esculentus. *AAPS PharmSciTech*.

[7] Builders P. F., Anwunobi P. A., Mbah C. C., Adikwu M. U. (2013). New direct compression excipient from tigernut starch: physicochemical and functional properties. *AAPS Pharmarmaceutical Science and Technology*.

[8] Zhu F., Yang X., Cai Y., Bertoft E., Corke H. (2011). Physicochemical properties of sweetpotato starch. *Starch - Stärke*.

[9] Craig S. A., Maningat C. C., Seib P. A., Hoseney R. C. (1989). Starch paste clarity. *Cereal Chemistry*.

[10] Coskuner Y., Ercan R., Karababa E., Nazlican A. (2002). Physical and chemical properties of chufa ( Cyperus esculentus L ) tubers grown in the Cukurova region of Turkey. *Journal of the Science of Food and Agriculture*.

[11] Simi K., Abraham T. (2008). Physicochemical rheological and thermal properties of njavara rice (oryza sativa ) starch. *Journal of Agricultural and Food Chemistry*.

[12] Lindeboom N., Chang P. R., Tyler R. T. (2004). Analytical, biochemical and physicochemical aspects of starch granule size, with emphasis on small granule starches: a review. *Starch - Stärke*.

[13] Cinnaswamy R., Hanna M. (1988). Relationship between amylose content and extrusion-expansion properties of con starches. *Cereal Chemistry*.

[14] Lee M. R., Swanson B. R., Baik B. K. (2001). Influence of amylose content on properties of wheat starch and breadmaking quality of starch and gluten blends. *Cereal Chemistry*.

[15] Bertoft E. (2017). Understanding starch structure: recent progress. *Agronomy*.

[16] Defloor I., Dehing I., Delcour J. A. (1998). Physico-chemical properties of cassava starch. *Starch - Stärke*.

[17] Tortoe C., Akonor P. T., Koch K., Menzel C., Adofo K. (2017). Amylose and amylopectin molecular fractions and chain length distribution of amylopectin in 12 varieties of Ghanaian sweet potato (Ipomoea batatas) flours. *International Journal of Food Properties*.

[18] Amani G. N. G., Tetchi F. A., Colonna P. (2007). Molecular and physicochemical characterisation of starches from yam, cocoyam, cassava, sweet potato and ginger produced in the Ivory Coast. *Journal of the Science of Food and Agriculture*.

[19] Tester R. F., Morrison W. R. (1990). Swelling and gelatinization of cereal starches. I. Effects of amylopectin, amylose, and lipids. *Cereal Chemistry*.

[20] Waterschoot J., Gomand S. V., Fierens E., Delcour J. A. (2015). Production, structure, physicochemical and functional properties of maize, cassava, wheat, potato and rice starches. *Starch - Stärke*.

[21] Gomand S. V., Lamberts L., Derde L. J. (2010). Structural properties and gelatinisation characteristics of potato and cassava starches and mutants thereof. *Food Hydrocolloids*.

[22] Kim Y. S., Wiesenborn D. P., Orr P. H., Grant L. A. (1995). Screening potato starch for novel properties using differential scanning calorimetry. *Journal of Food Science*.

[23] Singh N., Kaur L. (2004). Morphological, thermal, rheological and retrogradation properties of potato starch. *Journal of the Science of Food and Agriculture*.

[24] Karim A. A., Norziah M. H., Seow C. C. (2000). Methods for the study of starch retrogradation. *Food Chemistry*.

[25] Liu J., Wang B., Lin L. (2014). Functional, physicochemical properties and structure of cross-linked oxidized maize starch. *Food Hydrocolloids*.

[26] Zheng G. H., Sosulski F. W. (1998). Determination of water separation from cooked starch and flour pastes after refrigeration and freeze–thaw. *Journal of Food Science*.

[27] Noda T., Takigawa S., Matsuura-Endo C. (2005). Physicochemical properties and amylopectin structures of large, small, and extremely small potato starch granules. *Carbohydrate Polymers*.

[28] Eliasson A.-C., Karlsson R. (1983). Gelatinization properties of different size classes of wheat starch granules measured with differential scanning calorimetry. *Starch ‐ Stärke*.

[29] Asaoka M., Blanshard J. M. V., Rickard J. E. (1992). Effects of cultivar and growth season on the gelatinisation properties of cassava (manihot esculenta) starch. *Journal of the Science of Food and Agriculture*.

[30] Hoover R. (2001). Composition , molecular structure , and physicochemical properties of tuber and root starches: a review. *Carbohydrate Polymers*.

[31] Mua J. P., Jackson D. S. (1997). Relationships between functional attributes and molecular structures of amylose and amylopectin fractions from corn. *Journal of Agricultural and Food Chemistry*.

[32] Miles M. J., Morris V., Orford P., Ring S. (1985). The role of amylose and amylopectin in the gelation and retrogradation of starch. *Carbohydrate Research*.

[33] Bourne M. (2002). *Food Texture and Viscosity Concept and Measurement*.

[34] Nurul I. M., Azemi B. M. N., Manan D. M. A. (1999). Rheological behaviour of sago (Metroxylon sagu) starch paste. *Food Chemistry*.

